# Microcystin *mcyA* and *mcyE* Gene Abundances Are Not Appropriate Indicators of Microcystin Concentrations in Lakes

**DOI:** 10.1371/journal.pone.0125353

**Published:** 2015-05-06

**Authors:** Lucas J. Beversdorf, Sheena D. Chaston, Todd R. Miller, Katherine D. McMahon

**Affiliations:** 1 Department of Civil and Environmental Engineering, University of Wisconsin-Madison, 1415 Engineering Drive, Madison, Wisconsin, 53706, United States of America; 2 Department of Bacteriology, University of Wisconsin-Madison, 1550 Linden Drive, Madison, Wisconsin, 53706, United States of America; University of Connecticut, UNITED STATES

## Abstract

Cyanobacterial harmful algal blooms (cyanoHABs) are a primary source of water quality degradation in eutrophic lakes. The occurrence of cyanoHABs is ubiquitous and expected to increase with current climate and land use change scenarios. However, it is currently unknown what environmental parameters are important for indicating the presence of cyanoHAB toxins making them difficult to predict or even monitor on time-scales relevant to protecting public health. Using qPCR, we aimed to quantify genes within the microcystin operon (*mcy*) to determine which cyanobacterial taxa, and what percentage of the total cyanobacterial community, were responsible for microcystin production in four eutrophic lakes. We targeted *Microcystis*-16S, *mcyA*, and *Microcystis*, *Planktothrix*, and *Anabaena*-specific *mcyE* genes. We also measured microcystins and several biological, chemical, and physical parameters—such as temperature, lake stability, nutrients, pigments and cyanobacterial community composition (CCC)—to search for possible correlations to gene copy abundance and MC production. All four lakes contained *Microcystis-mcyE* genes and high percentages of toxic *Microcystis*, suggesting *Microcystis* was the dominant microcystin producer. However, all genes were highly variable temporally, and in few cases, correlated with increased temperature and nutrients as the summer progressed. Interestingly, toxin gene abundances (and biomass indicators) were anti-correlated with microcystin in all lakes except the largest lake, Lake Mendota. Similarly, gene abundance and microcystins differentially correlated to CCC in all lakes. Thus, we conclude that the presence of microcystin genes are not a useful tool for eliciting an ecological role for toxins in the environment, nor are microcystin genes (e.g. DNA) a good indicator of toxins in the environment.

## Introduction

In recent decades, there has been an apparent increase in the frequency and intensity of cyanobacterial harmful algal blooms (cyanoHABs) in freshwater lakes [1]. These cyanoHABs are unsightly resulting in decreased recreational and property value [2]. Upon decay they can deplete dissolved oxygen leading to fish die-offs. Some contain toxins, which impact ecosystem and public health. Microcystin is the most commonly detected and measured cyanobacterial toxin. Although the ecology of cyanobacteria is well described, the relationship between population dynamics and environmental triggers of microcystin production are poorly understood. At least 90 different structural isoforms of microcystin have been identified thus far [3], but little is known about their varying toxicity or how they might differentially affect children and adult development [4]. Microcystins bind to serine/threonine protein phosphatases in animal liver cells [5], eventually leading to tissue hemorrhaging. Acute exposure to microcystin has resulted in fatalities [1], and recent epidemiological studies suggest that chronic exposure to low concentrations of microcystin may lead to liver and colorectal cancer [6,7]. Thus, there is both a need to understand the underlying mechanisms of microcystin production and to develop technologies to better monitor and protect public health.

Microcystin is known to be produced by *Microcystis*, *Anabaena*, *Planktothrix* [8] and has been associated with several others [9]. It is synthesized via non-ribosomal peptide synthesis (NRPS) and polyketide synthase (PKS) modules. Ten genes (*mcyA-J*) are involved in microcystin production (55 kbp total) in *Microcystis*, *Anabaena*, and *Planktothrix* [10,11]. In *Microcystis*, these genes are found in the *mcy* operon and are bidirectionally transcribed from the *mcyA/D* central promoter [12]. In *Anabaena*, these genes are arranged in three operons [13], and in *Planktothrix*, the arrangement is identical to *Microcystis*. Two transcriptional start sites have been identified for *mcyA* and *mcyD*, dependent upon light intensities, with additional transcriptional start sites found in *mcyA*, *mcyD*, *mcyE*, *mcy*F, *mcyG*, *mcyH*, *mcyI and mcyJ* [12,14,15]. However, it is still unknown what environmental conditions trigger microcystin production [16] making them difficult to predict on short time-scales (e.g. daily-weekly).

Cyanobacteria are most often enumerated and identified using microscopic counts and pigment concentrations (e.g. chlorophyll and phycocyanin). This can be an adequate means of monitoring cyanobacterial biomass and coarse population dynamics, but is ineffective at monitoring microcystin-producing populations because only some members of a cyanobacterial genus contain genes capable of encoding for toxin biosynthesis. A toxic (or potentially toxic) cyanobacterium can appear morphologically identical to a non-toxic cyanobacterium (i.e. lacking a complete *mcy* gene cluster) of the same species [17]. Furthermore, toxic and non-toxic cyanobacteria are commonly found in the same cyanobacterial bloom [17]. The percentage of cells containing toxin genes may vary significantly over both space and time [18] and there is little understanding for what environmental factors control changes in the fraction of cells capable of producing toxin. In fact, few studies have examined the relationship between the percentage of *mcy*-containing organisms with actual microcystin concentrations in lakes over both spatial and temporal scales during the cyanobacterial growth season [19,20,21], but none to this extent.

In this study, four lakes in Dane County, Wisconsin, USA were sampled weekly throughout the summer and tested for the presence and abundance of cyanobacteria with and without microcystin genes using quantitative polymerase chain reaction (qPCR). In addition, microcystins were measured using liquid chromatography tandem mass spectrometry (LC-MS/MS). The purpose of this study was to examine the population dynamics of cyanobacteria that have the potential to produce microcystin over the course of a summer using molecular methods, determine if environmental factors can be used to predict the fraction of cells capable of producing microcystins, and establish whether the abundance of such cells relates at all to measured toxin concentrations.

## Materials and Methods

### Ethics statement

No specific permits were required for the described field study, nor were any specific permissions needed to sample the following locations. None of the lakes in this study were privately owned, and our sampling did not involve endangered or protected species.

### Sample Sites

Lakes Mendota, Monona, Wingra, and Kegonsa were sampled weekly from June 1-October 21, 2008. These lakes are located in Dane County, in south central Wisconsin near the city of Madison, and are all connected (either directly or indirectly) via the Yahara River within the Yahara Watershed (S1 Fig). All four lakes are highly eutrophic and suffer from chronic cyanoHABs [22], but they vary by size, depth, and surrounding landscape (Table 1) making them a compelling system for comparative studies. We sampled the deepest location (“Deep Hole”) of each lake, which ranged from 4 m in Lake Wingra to 25 m in Lake Mendota. We chose these locations for several reasons. First, previous studies in these lakes have shown that, while some spatial variability exists between deep and very shallow beach locations [23], within lake spatial variability is generally less than vertical heterogeneity [24,25]. Second, the Deep Hole locations of these lakes have been sampled biweekly since 1985 as part of the North Temperate Lakes-Long Term Ecological Research (NTL-LTER) program centered at the University of Wisconsin-Madison Center for Limnology. Third, the Lake Mendota Deep Hole location contains a moored buoy that collects high-resolution biological, physical, and meteorological data. Thus, we selected sample locations that were most representative of the pelagic area and contained historical, as well as high resolution, data.

**Table 1:** Characteristics of four eutrophic lakes sampled: Lakes Mendota, Monona, and Kegonsa are directly connected via the Yahara River, while Lake Wingra is primarily fed by ground water and urban run-off and runs into Lake Monona.

Lake characteristics	Mendota	Monona	Kegonsa	Wingra*
Coordinates	43°36'24" N, 89°25'24" W	43°4'9" N, 89°21'34" W	42°57'50" N, 89°14'60" W	43°3'13" N, 89°25'11" W
Area (km^2^)	39.4	13.3	13.0	1.3
Max depth (m)	25.3	22.6	9.8	4.3
Mean depth (m)	12.7	8.3	5.1	2.7
Water residence time (yr)	4.5	0.9	0.5	ND
Landscape	U, F, W	U, F	U, F, W	U, F, W
Primary non-point pollution	Ag	Ag	Ag	U

ND = not determined. U = urban, F = forested, W = wetland, Ag agriculture

*Lake Wingra is primarily fed by ground water and urban run-off

### Sampling Regime

Sampling was performed once or twice weekly from June-October for a total of 17–25 sample dates per lake. Sampling occurred at the deepest site in each lake (Deep Hole herein). First, Secchi disk depth was taken and multiplied by 1.7 [26] to estimate the photic zone depth (PZD). Then, integrated water samples were collected, using a PVC tube, from the surface down to the PZD and stored on ice in a cooler until transported back to the lab for analysis. Dissolved oxygen (DO), pH, and water temperature were measured through the entire water column at discrete 1 m intervals using a 556 MPS YSI meter (YSI, Yellow Springs, OH). Between 100–250 mL of lake water was filtered onto a 0.2 μm SUPOR filter (47 mm diameter; Pall Life Sciences, Ann Arbor, MI) and stored at -80°C in preparation for DNA extractions. For chlorophyll-*a* and phycocyanin quantification, 1 L of water for each was filtered onto a 47 mm glass fiber filter (nominal pore size 0.7 μm; GF/F; #1825–047; Whatman, England) and stored at -20°C until analyses were performed. The GF/F filtrate was collected and frozen at -20°C in preparation for soluble reactive phosphorus (SRP) and nitrate/nitrite analysis. Whole water samples (i.e. unfiltered) were collected and frozen at -20°C in preparation for total phosphorus (TP) and microcystin analysis.

### Analytical measurements

All analytical procedures used have been previously described [24]. Briefly, SRP was measured by the ascorbic acid-molybdenum blue method 4500 P E [27]. Nitrate and nitrite were measured individually using high-performance liquid chromatography (HPLC)[28] and reported individually, or as combined (N + N). TP was digested as previously described [29] and then determined as SRP. Phycocyanin was extracted in 20 mM sodium acetate buffer (pH 5.5) and measured spectrophotometrically following centrifugation [30]. Chl-*a* was extracted in 90% acetone and measured spectrophotometrically with acid correction [31].

For toxin analysis, whole water samples were lyophilized, resuspended in 5% acetic acid, freeze-thawed three times, separated by solid phase extraction (SPE; Bond Elut C18 column, Varian), and eluted in 100% methanol as previously described [32]. Microcystin (MC) variants of leucine (L) arginine (R), alanine (A), and tyrosine (Y) were detected and quantified at the Wisconsin State Laboratory of Hygiene (SLOH) using electrospray ionization-tandem mass spectrometry (API 3200, MS/MS) after separation by liquid chromatography (LC)[33,34]. All four microcystins were added and reported herein as total microcystin (MC).

### DNA extraction and polymerase chain reaction

DNA was extracted from filters using a xanthogenate-phenol-chloroform protocol previously described [35,36]. DNA pellets were resuspended in 100 μL of ddH_2_O. Resulting DNA concentrations were quantified using Picogreen (Invitrogen, Eugene, OR) and diluted to a concentration of 10 ng μL^-1^.

All samples were evaluated using end-point PCR for three separate genes using eight previously published primer sets (S1 Table). All PCR reactions were performed using iQ Super mix (Bio-Rad, Hercules, CA) on an Eppendorf thermocycler using previously published primer concentrations and thermocycler parameters [37,38,39,40]. PCR products were run on a 1% agarose gel stained with ethidium bromide to determine the presence or absence of each gene.

All qPCR reactions were performed using iQ SYBR green supermix (Bio-Rad, Hercules, CA) on an iCycler (Bio-Rad, Hercules, CA). Each sample was run in triplicate and each qPCR plate contained a duplicate six-point standard curve with values ranging from 10^3^–10^8^. Ten ng of sample DNA was added to each reaction. All qPCR was performed using previously published thermocycler parameters (S1 Table). Standards for each plate were generated by cloning PCR product from a microcystin-producing strain (University of Texas-Austin, strain UTEX 2385) into a vector using the TOPO TA cloning kit (Invitrogen, CA), extracting the recombinant plasmid using a QIAGEN plasmid mini kit (QIAGEN, Valencia, CA), cutting it with restriction enzyme *ScaI* (Promega, Madison, WI), purifying using QIAquick PCR purification kit (QIAGEN), and quantifying using a NanoDrop (Thermo Fisher Scientific, Waltham, MA). All plasmids were sequenced to confirm the inserts. All standard curves yielded R^2^ > 0.99 and qPCR efficiencies > 90%. At the end of each qPCR reaction, a melt curve was performed, as well as validation via ethidium bromide gel electrophoresis, to check for non-specific amplification. Copy numbers for each sample were calculated using an equation generated from the average of the standard curves in each plate. Copy numbers were also multiplied by a dilution factor and a water-filtering factor to present data as gene copies mL^-1^ of filtered lake water. In addition to this, the *Microcystis-*16S copy numbers were divided by two to account for multiple copies of the 16S gene observed in the *Microcystis aeruginosa* NIES-843 genome [41].

Cyanobacterial community composition was assessed using automated phycocyanin intergenic spacer analysis (APISA) as previously described [23,24]. Canonical correspondence analyses (CCA) were performed in the R Statistics Environment [42] Vegan Package [43] using a Bray-Curtis similarity matrix that was based on relative abundance data [44]. Prior to analysis, toxin and gene copy data were log transformed. All correlations were performed using the *envfit* function within the R Vegan Package.

### Lake stability calculations

Lake stability characteristics were calculated using the Lake Analyzer program previously described [45]. Briefly, Schmidt Stability (SS), Lake Number (L_N_), and metalimnion top (metaT) were estimated from water temperature profiles and meteorological data collected at the time of sampling, as well as lake bathymetry. SS represents the resistance to mixing due to the energy stored within the lake (i.e. how stratified the lake is) and L_N_ represents the amount of wind induced mixing within the lake. Very simply, both indices are expected to increase as a lake becomes more thermally stratified as it takes more energy and wind forcing to cause the water column to mix. Lake Analyzer also calculates the thermocline depth, as well as the top and bottom of the metalimnion. The metaT depth to the lake surface, as defined here, is synonymous with the upper mixed layer depth of the epilimnion.

### Within- and between-lake statistics

Pearson correlations were performed for in-lake comparisons to look for relationships between the abundance of the *Microcystis-*16S rDNA (*Mic-*16S), *mcyA*, and *Microcystis*-*mcyE* (*Mic-mcyE)* genes with each other and with all available environmental data. In addition, the gene copy number for *Mic-mcyE* was divided by the number of adjusted gene copies for the *Mic-*16S to estimate the proportion of *Microcystis* cells containing toxin genes in each lake for each sampling event. *Mic-mcyE* was also divided by the general *mcyA* to determine what proportion of the potentially toxic cyanobacterial population was *Microcystis*. Gene copy numbers and environmental parameters were log-transformed for statistical analysis. All comparisons were performed in MatLab [46] with calculated R and *p* values reported (including with or without Bonferroni Correction). We chose to use a Bonferroni Correction to reduce potential false positives that can result from multiple comparisons, as well as to focus on the strongest relationships that may exist. All between-lake comparisons were performed in MatLab using a one-way analysis of variance (ANOVA). Boxplots generated display the median and 25^th^ and 75^th^ percentiles. Boxes that have non-overlapping notches are considered significantly different (*p* values reported). Whiskers represent the minima and maxima, which are not considered outliers (± 2.7σ), and the symbols are the outliers.

## Results

### Within lake comparisons

Pair-wise comparisons of the three target genes in Lake Mendota yielded strong (≥ 0.92) Pearson correlation coefficients between *mcyA*, *Mic-*16S, and *Mic-mcyE* (S2 Table). Lake Monona yielded correlation coefficient values of a similar strength with (≥ 0.89) between *mcyA*, *Mic-*16S, and *Mic*-*mcyE* (S3 Table). Lake Kegonsa also yielded strong correlation coefficient values (≥ 0.98; S4 Table). Pair-wise correlation values for Lake Wingra were weaker, yielding 0.58 between *mcyA* and *Mic-*16S, 0.73 between *Mic-*16S and *Mic-mcyE*, and 0.82 between *Mic-mcyE* and *mcyA* (S5 Table). In Lakes Mendota, Monona, and Kegonsa, all *mcyA*, *Mic*-*mcyE*, and *Mic*-16S pairwise comparisons were significant at *p* < 0.0007 and Wingra at *p* < 0.05. Since *mcyA* was highly correlated to *Mic*-*mcyE* and *Mic*-16S, *Mic*-*mcyE* was highly correlated to *Mic*-16S, and there was a lack of *Anabaena* and *Planktothrix* detects (S6 Table), it is likely that MC production in these four eutrophic lakes originated from *Microcystis* only. We then proceeded to see which environmental parameters, if any, correlated with these genes.

**Fig 1:**
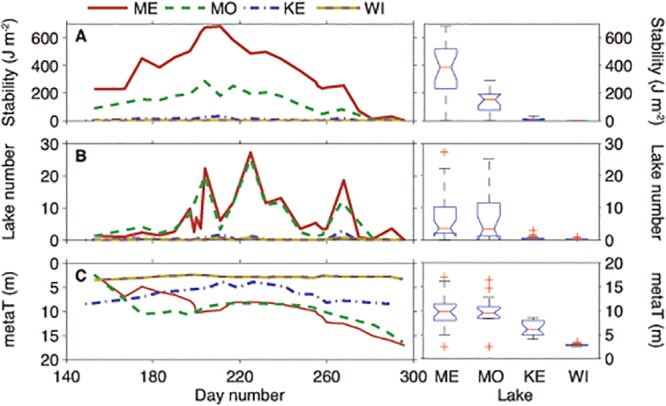
Time-series comparison of Schmidt Stability (SS), Lake Number (L_N_), and the top of the metalimnion (metaT), which comprises the upper mixed layer, as boxplot results of one-way analysis of variance (ANOVA) of variables between the lakes. Lakes are in order from largest to smallest; left to right: ME = Mendota, MO = Monona, KE = Kegonsa, and WI = Wingra.

**Fig 2:**
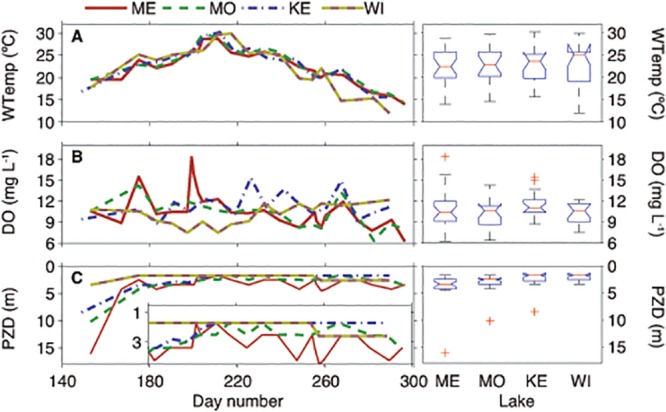
Time-series comparison of water temperature (WTemp), dissolved oxygen (DO), and photic zone depth (PZD), as well as boxplot results of one-way analysis of variance (ANOVA) of variables between the lakes. Lakes are in order from largest to smallest; left to right: ME = Mendota, MO = Monona, KE = Kegonsa, and WI = Wingra. PZD inset represents only days 180–300.

**Fig 3:**
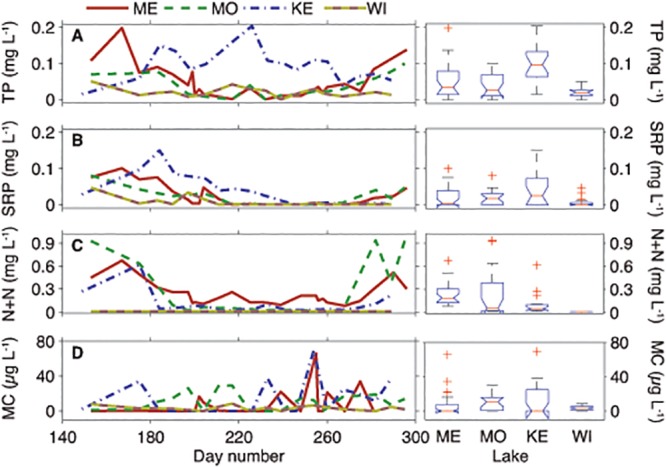
Time-series comparison of total phosphorus (TP), soluble reactive phosphorus (SRP), nitrate plus nitrite (N + N) and total microcystin (Total MC), as well as boxplot results of one-way analysis of variance (ANOVA) of variables between the lakes. Lakes are in order from largest to smallest; left to right: ME = Mendota, MO = Monona, KE = Kegonsa, and WI = Wingra.

In most cases, each of the four lakes exhibited characteristically strong seasonal gradients (i.e. increasing temperature and thermal stratification) with nutrient depletion throughout the sampling period (Figs 1–3, Table 2). Thus, we expected *mcyA*, *Mic*-*mcyE*, and *Mic*-16S to positively correlate to temperature, lake stability, increased biomass and nutrient depletion within a lake. In Lake Mendota, *Mic-*16S, *Mic*-*mcyE*, and *mcyA* were all significantly negatively correlated to SRP and positively correlated to metaT (*p* < 0.0007, S2 Table). Though not significant, all genes were strongly negatively correlated to Secchi and PZD as well. Weak negative correlations were detected between nitrate, nitrite, and TP and weak positive correlations were found with phycocyanin and MC. In Lake Monona, *mcyA*, *Mic*-*mcyE*, and *Mic*-16S were not significantly correlated to any environmental parameters (*p* < 0.0007; S3 Table). However, *mcyA* and *Mic*-*mcyE* trended with strong negative correlation to nitrate, nitrate + nitrite, TP, and PZD and positively with water temperature. Lake Kegonsa was the only lake in which TP and metaT increased during the summer (Figs 3 and 1, respectively). As such, *Mic*-*mcyE* and *mcyA* were significantly positively correlated to TP (*p* < 0.0007; S4 Table) and trended negatively (but not significantly) with metaT. *Mic*-*mcyE*, *mcyA* and *Mic*-16S also trended negatively, non-significantly, with Secchi and PZD and positively with water temperature. In Lake Wingra, *mcyA*, *Mic*-16S, and *Mic*-*mcyE* were not significantly correlated to any environmental parameters at a *p*-value of 0.0007 (S5 Table). Gene copies were the lowest and most variable in Lake Wingra as well (Table 3). However, *Mic*-16S trended positively with water temperature and SS and negatively with DO. *Mic*-*mcyE* also trended positively with DO and water temperature, but was only weakly correlated to all other environmental parameters measured.

**Table 2:** Average (range) environmental characteristics of the 4 eutrophic lakes sampled in this study from May-October. BDL = below detection limit.

Lake characteristics	Mendota	Monona	Kegonsa	Wingra*
Water temperature (°C)	22.4	22.8	22.8	22.5
	(14.0–28.7)	(14.6–29.7)	(15.6–30.2)	(12.0–29.9)
Dissolved oxygen (mg L^-1^)	10.8	10.3	11.4	10.1
	(6.3–18.3)	(6.4–14.3)	(8.7 15.4)	(7.5–12.2)
Photic zone depth (m)	3.7	3.1	2.4	2.0
	(1.7–16.1)	(1.7–10.2)	(1.7–8.5)	(1.7–3.4)
Total P (μg L^-1^)	50	40	100	20
	(BDL—190)	(BDL—100)	(15–200)	(BDL—50)
Soluble reactive P (μg L^-1^)	20	20	40	10
	(BDL—100)	(BDL—80)	(BDL—150)	(BDL—50)
Nitrate (μg L^-1^)	180	70	60	BDL
	(65–450)	(10–550)	(7–220)	
Nitrite (μg L^-1^)	60	180	40	BDL
	(BDL—230)	(5–920)	(BDL—420)	
Nitrate + Nitrite (μg L^-1^)	240	260	105	BDL
	(80–675)	(20–940)	(20–615)	
Chlorophyll-a (μg L^-1^)	54	107	43	128
	(BDL—210)	(BDL—250)	(BDL—240)	(BDL—230)
Phycocyanin (μg L^-1^)	104	107	112	112
	(4–560)	(6–290)	(2–370)	(BDL—290)
Total microcystins (μg L^-1^)	7.1	10.7	13.2	3.4
	(BDL—66)	(BDL—30)	(BDL—70)	(BDL—8.5)
Schmidt Stability (J m^-2^)	367	134	10.1	1.1
	(1.8–685)	(0.1–287)	(0.0–32)	(0–2.9)
Lake number (unitless)	6.7	6.7	0.5	0.2
	(0.0–27.3)	(0.0–25.3)	(0.0–2.9)	(0.0–0.9)
Metalimnion top (m)	9.8	9.9	6.3	2.8
	(2.5–17.1)	(2.5–16.4)	(4.0–8.5)	(2.4–3.5)

*Lake Wingra is primarily fed by ground water and urban run-off

**Table 3:** Summary qPCR statistics for each of the four lakes.

	Mendota	Monona	Kegonsa	Wingra
*Microcystis*-16S	100	100	330	20
	(BLQ—202)	(BLQ—256)	(BLQ—521)	(BLQ—31)
*McyA*	65	45	120	5
	(BDL—240)	(BLQ—180)	(BLQ—550)	(BDL—10)
*Mic-mcyE*	50	30	95	5
	(BDL—180)	(BLQ—120)	(BLQ—350)	(BDL—10)
*Mic*-*mcyE*/*Mic*-16S*	97%	74%	52%	21%
	(11–187%)	(15–142%)	(13–99%)	(0–52%)
*Mic*-*mcyE*/mcyA**	78%	82%	74%	4400%
	(16–138%)	(52–127%)	(37–95%)	(54–44000%)

Values are expressed as the number of gene copies x 10^3^ mL^-1^ of lake water filtered. BDL = below detection limit. BLQ = below level of quantification.

*Assuming two 16S gene copies cell^-1^ (Kaneko *et al*., 2007), *Mic-mcyE/Mic*-16S represents the percentage of the *Microcystis* community that is toxic.

**Mic-mcyE/mcyA represents the percentage of the toxic community that is *Microcystis*

Contrary to gene copy abundance, MC concentrations did not significantly correlate with any genes or environmental parameters in any of the lakes (*p* < 0.0007; S2–S5 Tables). Again, this underscores how toxin concentrations, and the conditions that constrain toxin production, vary on daily to weekly time-scales. MC concentrations were highest in Lakes Monona and Kegonsa, and MC trended with measured parameters in only those lakes. In Lake Monona, MC concentrations were negatively correlated to *mcyA*, *Mic-mcyE*, and *Mic*-16S at a *p*-value of 0.05 (i.e. without Bonferroni correction). Also, MC concentrations trended negatively with water temperature, TP, SRP in Lake Kegonsa and positively with nitrate, N + N, and metaT (all *p* < 0.05).

### Between lake comparisons

All 4 lakes in this study were eutrophic and similar biologically and chemically (Table 2). Physically, the lakes were quite different due to their varying size, shape, and maximum depth. All lakes were significantly different (*p* << 0.01) in terms of thermal stability (i.e. SS; Fig 1) with the deeper lakes exhibiting stronger thermal stratification as expected. However, Lakes Monona and Mendota were not significantly different from each other (*p* > 0.9) by Lake Number (L_N_), which incorporates wind shear, nor were Lakes Kegonsa and Wingra significantly different from each other (*p* > 0.1). By this measure, Mendota and Monona were significantly more stable than Kegonsa and Wingra (*p* < 0.01). Additionally, the low SS and L_N_ values for Lakes Kegonsa and Wingra suggest that they were polymictic in 2008. MetaT in all lakes was significantly different (*p* < 0.01) from one another, except in comparison of Lakes Mendota and Monona (*p* > 0.9; Fig 1). As stated earlier, metaT increased throughout the summer in polymictic Lake Kegonsa, whereas in Lakes Mendota and Monona it decreased throughout the summer as expected with warming upper-mixed-layer waters and a deepening of the thermocline.

Despite the varying thermal stability conditions, water temperature, DO, and PZD were not significantly different by a one-way ANOVA between the lakes (*p* > 0.01, Fig 2). If the June sampling dates are removed however (*see* Fig 2 inset), PZD variability significantly decreases and Lakes Mendota (mean ~ 3.4 m) and Monona PZD (mean ~ 2.7 m) both become significantly greater (*p* < 0.01) than Lakes Kegonsa (mean ~ 2.5 m) and Wingra (mean ~ 1.0 m), albeit by ~ 2 m. Additionally, the photic zone did extend through the entire water column in Lake Wingra in some cases. Nutrients were lowest in Lake Wingra, and both nitrate and nitrite were below detection for the entire sampling period (Fig 3). This may be a result of being fed by urban storm water run-off and groundwater, and/or the large number of macrophytes in Lake Wingra, whereas Lakes Mendota, Monona, and Kegonsa are larger and receive heavy nutrient loading from agricultural run-off from the Yahara Watershed. On average, TP and SRP were higher in Lake Kegonsa (TP significantly; *p* < 0.05), whereas nitrate was highest in Lake Mendota.

Interestingly, average phycocyanin concentrations were greatest in Lake Wingra, despite having the lowest nutrients, though phycocyanin concentrations were not significantly different between the four lakes throughout the sampling period (*p* > 0.99; S2 Fig). Chl-*a* was also highest in Lake Wingra, and was significantly greater than all other lakes (*p* < 0.01). Lake Monona chl-*a* was also significantly greater than that of Lakes Mendota and Kegonsa (*p* < 0.05). Conversely, chl-*a* was lowest in Lake Kegonsa despite having the highest TP and SRP concentrations.

There were common trends among the different target genes within the various lakes, with the exception of Lake Wingra, in that they were low in spring, peaked in mid-summer, and then declined into autumn (S3 Fig; Table 3). However, gene copy numbers were highly variable throughout the sampling period, demonstrating the importance of making multiple measurements through time. On average, Lake Kegonsa had the highest number of *mcyA*, *Mic*-*mcyE*, and *Mic*-16S gene copy numbers across the sampling period, though *mcyA* and *Mic*-*mcyE* were not significantly different between Lakes Kegonsa and Mendota (*p* > 0.05). In all other cases, Lake Kegonsa gene copy numbers were significantly greater than the other lakes (*p* < 0.05). Lake Wingra had the lowest number of all genes, which were significantly lower than all other lakes (*p* < 0.05). Lake Mendota and Lake Monona did not have significantly different copy numbers of any of the measured genes (*p* > 0.5).

**Fig 4:**
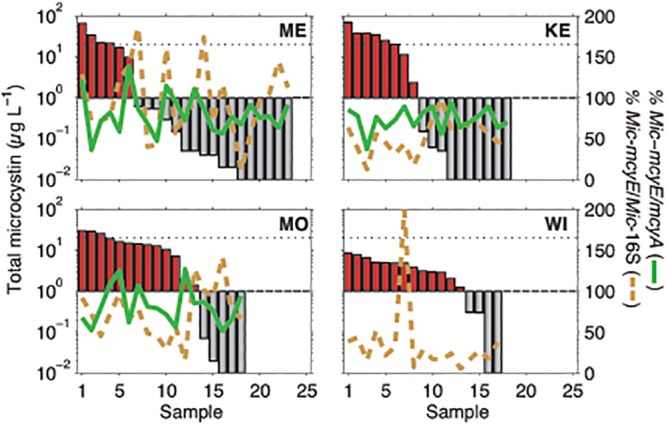
Comparison of percent *Microcystis*-*mcyE*/*mcyA* and percent *Microcystis*-*mcy*/*Microcystis*-16S versus the concentration of total microcystin observed (bars). For correlation coefficients, refer to the text or S2–S5 Tables. The black dashed line is the World Health Organization (WHO) level for safe drinking water (1 μg L^-1^), while the dotted line is the WHO level for moderate recreational risk (40 μg L^-1^). Note that Lake Wingra had the most detects above 1 μg L^-1^, despite having the lowest concentrations of Total MC and percent toxic *Microcystis* observed.

We defined *Mic*-*mcyE* divided by *mcyA* (*Mic-mcyE*/*mcyA*) as the proportion of potential microcystin producers that were *Microcystis*, since the *mcyA* primers we used should encompass *Microcystis*, *Planktothrix*, and *Anabaena*, while the *Mic-mcyE* primers target only *Microcystis*. In Lakes Mendota, Monona, and Kegonsa, the average *Mic*-*mcyE*/*mcyA* percentages were 78%, 82%, and 74%, respectively, and were not significantly different across lakes (Table 3; Fig 4). In Lake Wingra, the average *Mic*-*mcyE*/*mcyA* percentage was 4400% and was likely due to gene copy numbers that were near the detection limit. In any case, all of the existing data suggest that the majority of microcystin producers in these lakes were *Microcystis*. Furthermore, no *Anabaena*-*mcyE* was detected in 2008, and *Planktothrix*-*mcyE* was detected by only end-point PCR later in the season (S5 Table). We then defined the proportion of the *Microcystis* community that was potentially toxic by dividing *Mic*-*mcyE*/*Mic*-16S (Table 3; Fig 4). In rank order, lakes with higher potentially toxic *Microcystis* were Mendota (97%), Monona (74%), Kegonsa (52%) and Wingra (21%), with Wingra being significantly lower than all other lakes (*p* < 0.01). Lakes Mendota and Monona were also significantly greater than Lake Kegonsa (*p* < 0.01 and 0.05, respectively).

MC was highly variable across all four lakes (Fig 3). On average, Lake Kegonsa had the highest average MC concentration (13.2 μg L^-1^; Table 2), and the highest observed concentration on a single day (69 μg L^-1^; Fig 4). Lake Wingra had the lowest average MC concentration (3.4 μg L^-1^), which was significantly different from Lakes Monona (10.7 μg L^-1^) and Kegonsa (*p* < 0.05). However, Lake Wingra also had the highest percentage of samples above 1 μg L^-1^ (78%; Fig 4), the World Health Organization level for safe drinking water. The low MC concentrations in Lake Wingra are consistent with the low *Mic*-16S, *mcyA*, and *Mic*-*mcyE* gene copy numbers, but may also suggest that MC is constitutively expressed at low levels even when cell numbers are relatively low. Interestingly, while Lake Mendota had a potentially toxic *Microcystis* population of nearly 100%, the average MC concentration (7.1 μg L^-1^) was less than that of Lakes Kegonsa and Monona, in which *Mic*-*mcyE*/*Mic*-16S populations were roughly 50% and 75%, respectively. However, it should be noted that the overall MC comparisons across all lakes were not significantly different due to large temporal variation within lakes (*p* > 0.3).

### Variability in toxin genes and total microcystin with cyanobacterial community composition

**Fig 5:**
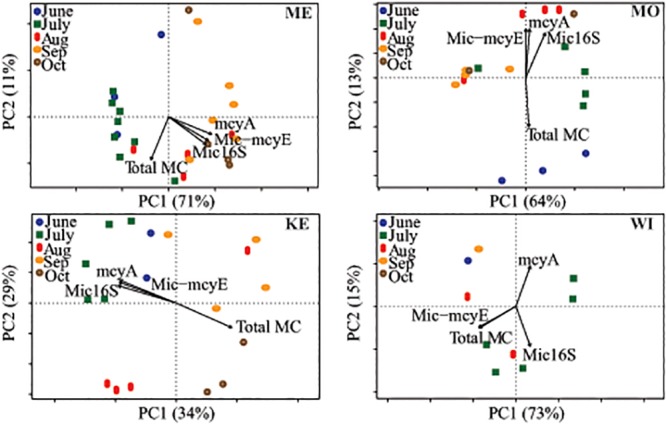
Canonical correspondence analysis (CCA) of cyanobacterial community composition (CCC) with toxin gene abundance and Total MC concentrations. The direction of the arrow indicates a positive correlation to those samples with the length representing the strength of correlation. In Lake Mendota, all toxin genes (*p* < 0.001) and Total MC (*p* < 0.05) were significantly correlated to CCC. In Lake Monona, only *mcyA* (*p* < 0.01) was significantly correlated to CCC. No significant correlations existed for Lakes Kegonsa and Wingra. Axes percentages indicate that amount of variability explained by that axis. ME = Mendota, MO = Monona, KE = Kegonsa, and WI = Wingra.

We asked whether variability in toxin gene abundance or MC concentration was correlated with variability in cyanobacterial community composition (CCC). In Lake Mendota, *mcyA*, *Mic-mcyE*, *Mic-*16S, and MC (R^2^ = 0.62, 0.61, 0.55, and 0.32, respectively) were all significantly correlated to changes in CCC (*p* < 0.05; Fig 5). However, MC was mostly correlated to changes in CCC that occurred earlier in the summer, whereas toxin gene abundance correlated to CCC much later in season when gene copy numbers peaked. In Lake Monona, only *mcyA* was significantly correlated to CCC (R^2^ = 0.47, *p* < 0.01), and all toxin gene abundances were anti-correlated to MC, which was most correlated with spring/early summer samples. Similarly, all toxin gene abundances were anti-correlated to MC in Lake Kegonsa. Though none of the variables were significantly correlated to CCC, an opposite trend was observed in Lake Kegonsa (compared to Lakes Monona and Mendota) where MC was most correlated to late summer/fall CCC and toxin gene abundances correlated to early summer CCC. Toxin gene abundances and MC were lowest in Lake Wingra and no significant relationships were observed with CCC (*p* > 0.25). In all lakes, toxin gene abundance did not follow the same trend with CCC, as did MC. Thus while toxin gene abundance is clearly related to the abundance of *Microcystis*, the influence of CCC on MC production is not simply due to the dominance of *Microcystis* in the population.

## Discussion

### Microcystis as the dominant microcystin producer

One of the initial goals of this study was to determine which cyanobacterial genera were capable of producing microcystin in four eutrophic lakes. These lakes were chosen because of their similar landscape and connectivity, they all suffer from noxious cyanoHABs, and they vary in shape and size. The *mcyA* primer set published by Hisbergues *et al*. [37] was designed to target all three of the major microcystin-producing genera: *Microcystis¸ Anabaena*, and *Planktothrix*. We used this primer set to gain an overall view of the cyanobacterial community members with the potential to produce microcystins. In addition, three primer sets were used to target the *mcyE* gene [39,47] of each specific microcystin-producing genus in order to better differentiate among the genera potentially involved in toxin production. *Mic-mcyE* and *mcyA* were detected via end-point PCR in all samples collected between June-October 2008 (S1 Table). *Planktothrix-mcyE* was detected via end-point PCR analysis in only two of the four lakes, Monona and Kegonsa, late in the season (September and October), and no *Anabaena-mcyE* was detected throughout the sampling period (S6 Table). Thus, taken together, the strong correlation between *Mic-mcyE* and *mcyA* (0.82, 1.00, 0.98, and 0.97 in Wingra, Kegonsa, Monona, and Mendota, respectively), the high *Mic*-*mcyE*/*mcyA* percentages, and the small number of times *Planktothrix* was detected, suggests that *Microcystis* was indeed the dominant microcystin producer in the four lakes studied.

At least 90 different cyanobacterial taxa were detected in these lakes forming a diverse community potentially influencing toxin levels. Variation in CCC was significantly correlated with toxin gene abundance and MC concentration in some lakes, but these two variables did not correlate with CCC in the same direction. Toxin gene abundance was correlated with the abundance of *Microcystis*, which explains the significant correlation between toxin gene abundance and CCC. Yet, MC levels correlated with CCC in an opposite direction in a canonical correspondence analysis. This indicates that MC concentration is not simply related to the abundance of the potentially dominant toxin producer.

### Microcystin correlations with the mcy genes

*Microcystis aeruginosa* appears to be the most common microcystin producer of this genus and sequencing efforts have revealed that *Microcystis aeruginosa* may contain two copies of the 16S rRNA gene [41]. A study revealed that among *Microcystis* isolates from a lake, 73% of *Microcystis aeruginosa* contained the *mcyB* gene, whereas only 17% of *Microcystis ichtyoblabe* and no *Microcystis wesenbergii* contained the *mcyB* gene [48]. In addition, *Microcystis aeruginosa* likely carries only one copy of the *mcy* genes [12]. None of the genes monitored—*Mic*-16S, *Mic*-*mcyE*, and *mcyA*—were significantly correlated to MC concentrations (following Bonferroni Correction; S2–S5 Tables). The same observation has been made in other systems [49,50], while at the same time some studies have shown positive correlations with these variables. We conclude that at least within these lakes, MC concentration varies with time and environmental conditions to such a degree that simple measures of gene copy numbers cannot explain the variation, particularly at a fixed sampling point. Additionally, there were some sampling dates in Lakes Mendota, Monona, and Wingra when the number of *Mic*-*mcyE* gene copies exceeded the corrected number of *Mic*-16S. Although it is theoretically impossible for the number of toxigenic *Microcystis* to exceed the number of total *Microcystis*, this has been observed in similar studies [51,52] and was viewed as an indication of extreme dominance of *Microcystis* carrying toxin genes in relation to the entire *Microcystis* community. It also reflects uncertainty about the actual gene copy number per genome in native populations, as compared to sequenced genomes. Finally, differential primer specificity and limitations to quantification using qPCR may also explain these unreasonably high ratios.

### Microcystin correlations with environmental parameters

MC concentrations did not significantly correlate (following Bonferroni Correction) to any environmental parameter in the four study lakes. Prior to Bonferroni Correction, only Lake Kegonsa had significant correlations with MC, which was strongly correlated to nitrate, nitrate + nitrite, SRP and TP, water temperature, and metaT. We note that Lake Kegonsa was an exception in this study in that TP increased during the summer and metaT decreased, which could have been caused by particle resuspension due to lake mixing (supported by the low Schmidt Stability and Lake Number; Fig 1). However, water temperature, which was nearly identical in the four lakes, was significantly negatively correlated to MC in Lake Kegonsa. Water temperature in Lake Kegonsa was above 15°C throughout the study period, which is generally considered the minimum growth temperature for *Microcystis*. However, on days when the temperature was above 25°C, microcystin concentrations were below the limit of detection, which is not consistent with prior studies citing increased microcystins with increasing temperature [53]. Furthermore, while the photic zone water temperature in all four lakes was essentially the same throughout this study (Fig 2), MC was very different. Thus, despite the multiple effects temperature can have on the increase in cyanobacterial dominance [54], temperature cannot be the sole controlling factor in cyanobacterial growth or toxin production in eutrophic lakes. Therefore, while climate projections may suggest an increase in cyanobacterial dominance due to rising temperatures, this does not necessarily translate to an increase in toxin production.

To our knowledge, no studies have measured both MC concentrations and toxin genes, both temporally and spatially, in multiple lakes at weekly time scales. Rinta-Kanto et al. [51] did an extensive 3 year study in the Lake Erie Western Basin and observed that microcystin, total phosphorus, *mcyD*, and *Microcystis*-16S were all positively correlated on monthly time scales. In our study in Lake Mendota, MC significantly positively correlated to *mcyA*, *Mic*-*mcyE*, and *Mic*-16S but significantly negatively correlated to Chl-*a* (R^2^ = 0.07, 0.74, 0.87, and 0.84, respectively; *p* < 0.05) on average monthly time-scales. However, on monthly time-scales in Lake Kegonsa, MC significantly negatively correlated to *mcyA*, *Mic-mcyE*, *Mic*-16S, water temperature, SS, and positively correlated to metaT (R_2_ = 0.82, 0.94, 0.96, 0.89, 0.89, and 0.73, respectively; *p* < 0.05). And, in Lake Wingra, only phycocyanin correlated to MC, strongly negatively (R^2^ = 0.94; *p* < 0.05). No parameters were correlated to MC on monthly scales in Lake Monona, and no nutrients (e.g. N and P or N: P ratios) correlated with MC in any lake. Thus, depending on the lake and time-scale for which the samples were collected, different parameters could be strongly correlated, weakly or negatively, or not correlated at all. Therefore, any studies aimed at using gene abundance as a predictor of toxin production in near real time, or studies aimed at modeling future scenarios, might misrepresent the extent of toxin production based solely on correlations.

An unresolved issue in cyanotoxin production is the mechanisms for which cyanotoxins (including microcystin and other secondary metabolites) are triggered in the environment. In culture-based studies, gene abundance and MC production have been related to N and P [55,56,57], iron (Fe)[15,58], sulfur (S)[59], light [8], growth rates [60,61], and nitrate uptake rates [62], as well as N stress [63] and C and N metabolism [64]. In the environment however, direct relationships are confounded by uncontrolled conditions and complex community interactions, but have been linked positively to P [65,66] and light [66,67] and negatively to N [24,68]. In our four study lakes, lake size played an interesting role. In lakes that were thermally stable (Mendota and Monona), gene copy numbers and biomass increased with temperature and nutrient depletion, as one might expect. In Lakes Kegonsa and Wingra, which are likely polymictic, that trend was less pronounced. Lake Mendota, the largest of the four lakes, had the highest percentage of toxic *Microcystis* (mean of 97%) but the lowest detects of MC concentrations above 1 μg L^-1^. Conversely, the smallest lake, Lake Wingra, had the lowest percentage of toxic *Microcystis* but the highest number of detects above 1 μg L^-1^. In Lake Wingra, nitrate and nitrite were both below detection for the entire sampling period. In a previous study, we suggested the N stress might stimulate toxin production in eutrophic lakes [24]. In Lake Wingra, MC production may be constitutively expressed due to the low amount of combined N available. However, because of the low biomass, as indicated by *Mic*-16S abundance, extremely high concentrations of MC couldn’t be obtained (Fig 4). In Lake Mendota, nitrate was the highest but MC concentrations were compared to Lakes Monona and Kegonsa, which had less nitrate. Thus, nitrogen may play an important, albeit complex, role in these lakes in both stimulating toxin production (deplete N) and for growth (replete N).

Toxin genes, biomass indicators, and toxin concentrations measured in this study were highly variable temporally. Additionally, there are several limitations to using gene quantification when compared to actual toxin measurements. It is possible for cyanobacteria to contain the target gene of interest, and therefore yield a positive result, but not produce toxin. This could be due to the absence of one or more of the required *mcy* genes via gene deletion, recombination, or transformation or due to gene disruption and inactivation by transposons or phage [69,70]. Second, qPCR of DNA provides little information about the levels at which that gene is being expressed and whether, or how much of, the toxin is being produced. Based on this study, we conclude that quantification of toxin gene abundance is unlikely to provide data leading to a mechanistic or predictable ecological model of MC production in lakes. We suggest that future studies focus on measuring the *expression* of toxin genes, along with toxin concentrations, at higher resolution and include lake physics to elucidate the role of toxins in the freshwater systems.

## Supporting Information

S1 Fig Map of the Yahara Lakes chain, which includes Lakes Mendota, Monona, and Kegonsa and Lake Wingra. Lakes Mendota, Monona, and Kegonsa are all connected via the Yahara River. Lake Wingra is fed by ground water and urban run-off and feeds into Lake Monona.
(EPS)

S2 Fig Time-series comparison of chlorophyll-*a* (Chl-*a*) and phycocyanin, as well as boxplot results of one-way analysis of variance of variables (ANOVA) between the lakes. Lakes are in order from largest to smallest; left to right: ME = Mendota, MO = Monona, KE = Kegonsa, and WI = Wingra.
(EPS)

S3 Fig Log transformed time-series comparison of *Microcystis*-16S, *Microcystis-mcyE*, and *mcyA*, as well as boxplot results of one-way analysis of variance of variables (ANOVA) between the lakes. Lakes are in order from largest to smallest; left to right: ME = Mendota, MO = Monona, KE = Kegonsa, and WI = Wingra.
(EPS)

S1 Table Primer sets used for PCR and qPCR analyses. (EPS)

S2 Table Pearson correlation coefficients for Lake Mendota (R values). Bold values indicate significant correlations (*p* < 0.0007) **after** Bonferroni Correction and italics indicate significant correlations (*p* < 0.05) *before* Bonferroni correction.
(EPS)

S3 Table Pearson correlation coefficients for Lake Monona (R values). Bold values indicate significant correlations (*p* < 0.0007) **after** Bonferroni Correction and italics indicate significant correlations (*p* < 0.05) *before* Bonferroni Correction.
(EPS)

S4 Table Pearson correlation coefficients for Lake Kegonsa (R values). Bold values indicate significant correlations (*p* < 0.0007) **after** Bonferroni Correction and italics indicate significant correlations (*p* < 0.05) *before* Bonferroni Correction.
(EPS)

S5 Table Pearson correlation coefficients for Lake Wingra (R values). Bold values indicate significant correlations (*p* < 0.0007) **after** Bonferroni Correction and italics indicate significant (*p* < 0.05) correlations *before* Bonferroni Correction. No correlations were calculated for nitrate, nitrate and N + N because no concentrations were above the limit of detection.
(EPS)

S6 Table Presence/absence end-point PCR for detection of the general *mcyA* primers and *mcyE* primers specific for *Microcystis* and *Planktothrix*. ND = no data.
(EPS)
